# Engineering Characteristics and Microscopic Mechanism of Soil–Cement–Bentonite (SCB) Cut-Off Wall Backfills with a Fixed Fluidity

**DOI:** 10.3390/ma16144971

**Published:** 2023-07-12

**Authors:** Tan Zhou, Jianhua Hu, Taoying Liu, Fengwen Zhao, Yanjun Yin, Mengmeng Guo

**Affiliations:** 1 School of Resources and Safety Engineering, Central South University, Changsha 410083, China; zt3153@csu.edu.cn (T.Z.); taoying@csu.edu.cn (T.L.); zhaofengwen@csu.edu.cn (F.Z.); yyj3378@csu.edu.cn (Y.Y.); 215511069@csu.edu.cn (M.G.); 2 Zijin School of Geology and Mining, Fuzhou University, Fuzhou 350108, China

**Keywords:** SCB cut-off wall, engineering characteristics, microscopic mechanism

## Abstract

Soil–cement–bentonite (SCB) backfill has been widely used in constructing cut-off walls to inhibit groundwater movement in contaminated sites. This study prepares SCB backfill with fixed fluidity. We conducted a series of experiments to investigate the engineering characteristics and microscopic mechanism of the backfill. The results indicate that the water content in the slurry was more sensitive to the bentonite content. The unconfined compression strength (UCS) value increased with an increase in the cement content, and the change with an increase in bentonite content was not noticeable. The permeability coefficient decreased distinctly with an increase in the cement and bentonite contents. The porosity of the SCB backfill increased with increasing bentonite content and decreased with increasing cement content. The UCS of SCB backfill was linearly and negatively correlated with the porosity; the permeability coefficient was not significantly related to the porosity. The percentage of micro- and small-pore throats in the backfill increased with increasing bentonite and cement contents. As cement and bentonite content increased by 6% in the backfill, the proportion of micro- and small-pore throats increased by 0.7% and 1.2%, respectively. The percentage of micro- and small-pore throats is deduced to be more suitable as a characterization parameter for the permeability of the SCB backfill. The overall results of this study show that the reasonably proportioned SCB backfill has potential as an eco-friendly and cost-effective material. Based on the requirements of strength and permeability coefficient (UCS > 100 kPa, 28 days permeability coefficient <1 × 10^−7^ cm/s), we suggested using a backfill with 12% bentonite and 9% cement as the cut-off wall mix ratio.

## 1. Introduction

Owing to economic factors, some large metal smelters have been placed in the inner parts of urban areas over the past decades. During the storage, transfer, and smelting of production materials in workshops, there is a high possibility of pollutants seeping into the site soil. Contaminants accumulate in the soil after contacting groundwater and gradually migrate with the contamination plume. The migration of contaminants can damage the environment and threaten the safety and health of humans [1,2,3]. Therefore, measures must be taken to limit groundwater migration and prevent the spread of contamination in anticipation of better treatment of contaminated sites [4,5].

Currently, cut-off walls are a popular geotechnical engineering method to remediate contaminated sites [6,7,8]. Soil–bentonite (SB) and cement–bentonite (CB) cut-off walls are widely used worldwide to retard the movement of groundwater and the migration of contaminant plumes by cutting off the flow of groundwater [9,10]. The currently available research on these two materials has focused on their permeability properties [11,12,13,14]. Morandini and Leite observed that the cation exchange capacity and plasticity of SB slurries increased with increasing bentonite proportion, while the permeability decreased [15]. In addition, the permeability coefficient suddenly decreased when the bentonite was increased to a larger proportion [16]. In general, the permeability coefficient of traditional CB backfill can reach between 10^−6^ and 10^−5^ cm/s, which is much higher than that of SB backfill (<10^−7^ cm/s) [17]. Wu et al. showed that the use of ground granulated blast furnace slag (GGBS) instead of cement significantly reduced the permeability coefficient of CB barriers with GGBS substitution ratios of up to 60–80% [18]. Similarly, some researchers used fly ash to enhance the impermeability of CB cut-off walls and found that the permeability coefficient of the cut-off wall material could reach 10^−8^ cm/s. It has been reported that the permeability coefficient of a fly ash-modified CB slurry is inversely and directly proportional to the fly ash admixture [19]. In terms of strength, studies have shown that the SB slurry is generally not strong and has a poor load-bearing capacity and interference resistance [20]. After construction, the primary consolidation effect contributes to the initial shear strength of the slurry, and the thixotropic properties and secondary consolidation effects of bentonite contribute to a further increase in strength, with an undrained shear strength of approximately 5–15 kPa [21,22]. The strength of the CB slurry is generally higher than that of the SB slurry. According to previous studies, the strength of a CB slurry was determined using the contents of the cement, GGBS, fly ash, and other cementitious materials in the slurry and the water–binder ratio (W/B). In some combinations, the unconfined compressive strength (UCS) of CB slurries can reach 1 MPa [23]. Unlike SB backfill, CB backfill material is prepared without the use of on-site soil. This is advantageous if the soil at the industrial site is contaminated. In addition, CB backfill is more suitable for use for complex and special needs, especially for sites with high mechanical strength requirements or low soil quality [24]. However, it is more expensive and not as impermeable as the SB backfill. Both materials have advantages and disadvantages. Because of the need for redevelopment after the remediation of industrial contaminated sites, the cut-off wall in use is subjected to long-term external disturbances. Therefore, the cut-off wall material must have both good impermeability and strength [25].

The soil–cement–bentonite (SCB) cut-off walls is a self-hardening slurry [26]. Compared to SB and CB cut-off walls, SCB walls have sufficient strength to accommodate surface loads [27]. Additionally, they can reach low permeation levels (<10^−7^ cm/s), and they have an extra economic advantage in that they use soils excavated on site [28,29]. To further reduce the cost of SCB slurries and improve their engineering properties, some researchers have conducted exploratory experiments on SCB cut-off walls. These tests focused on improving engineering performance after cement substitution using geopolymer materials [27,30]. Opdyke and Evans showed that the UCS varied significantly when the slag substitution ratio was below 60%, and reached a maximum at a substitution ratio of 80% [28]. Talefirouz found that the infiltration coefficient of SCB backfill was much lower when more than 50% of slag replaced cement [31]. There are relatively few studies on the soil components in SCB slurries, with a focus on specific soils that can increase strength or significantly reduce permeability. Wu et al. used sandy clay (sand content >80%) as the main component of an SCB backfill, wherein the UCS value of each ratio of the CSB materials, which were cured for more than 28 days, exceeded 500 kPa. The permeability coefficients were lower than 10^−8^ cm/s, and good bearing performance and impermeability were exhibited [32]. Li et al. used a sample with 46.66% clay content to prepare two groups of SCB slurries with clay contents of 95 and 94%, respectively. Both groups of materials had permeability coefficients below 2 × 10^−8^ cm/s after 7 days of curing [33]. It is noteworthy that the strength and permeability of SCB slurries depend on both the bentonite and cementitious material, and the water content in the slurry. The construction of SCB cut-off walls has high requirements for slurry fluidity in actual projects. Construction requires a slurry slumping range of 100–150 mm [34]. In practical engineering, a fixed slurry fluidity can significantly affect the initial water content of different proportions of materials, as bentonite and cement are highly absorbent. Therefore, according to the actual requirements of contaminated industrial sites and based on a large number of previous studies, it is necessary to perform a multi-scale characterization of SCB cut-off wall materials and to analyze their complex interactions in the context of determining slurry fluidity.

The fluidity of the slurry has a significant impact on the quality of the cut-off wall in practice. Higher water content enhances the slurry’s fluidity, facilitating construction but compromising the cut-off wall’s performance. Conversely, lower water content improves the cut-off wall’s performance but poses more challenges in construction. Most previous studies have assumed a constant moisture content of the slurry to examine the effects of other factors. However, such materials may not always be suitable for on-site construction.

Therefore, in this study, an SCB backfill with the same slump was prepared. The effects of different slurry ratios on the initial moisture content of the material were analyzed at the same slurry slumping. We studied how different cement and bentonite contents and curing time affected the engineering characteristics (UCS and permeability) of SCB backfill. In addition, a combination of nuclear magnetic resonance (NMR) tests and scanning electron microscopy (SEM) revealed the effects of the curing age and each major component on the micromorphology and pore structure of the SCB backfill. Finally, the relationship between the pore structure and engineering properties of the SCB backfill was analyzed and discussed, and the relationship between the pore structure and engineering properties was quantitively studied.

## 2. Experimental Program

### 2.1. Materials

**Figure 1:**
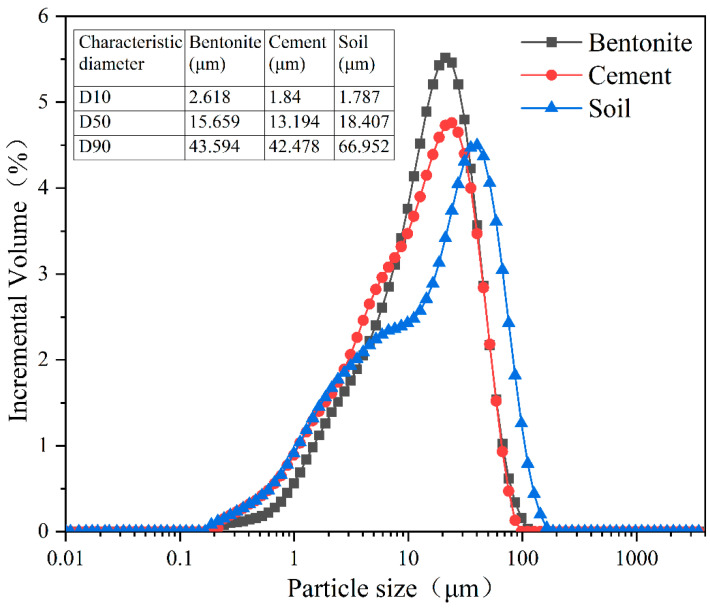
Particle size distributions of the soil, cement, and bentonite.

SCB backfill materials were prepared using soil, commercial bentonite, and cement. The experimental soil is widely distributed in the south-central region of China. The commercial bentonite is from a supplier in Shandong province. The cementitious material is ordinary 42.5 strength cement. Normal tap water was used for the experimental water. Their mineralogical properties and particle size distribution are shown in Table 1 and Figure 1, respectively.

Particle size analysis showed that soil had a characteristic median particle size Dv(50) of 18.407 μm. Soil had a low coefficient of curvature (<1), indicating poor gradation. Cement had a characteristic median particle size Dv(50) of 13.194 μm, indicating good gradation. Bentonite had a characteristic median particle size Dv(50) of 15.659 μm, indicating good gradation.

### 2.2. Specimen Preparation and Backfill Mix Design

First, specific amounts of bentonite and water were mixed and stirred for 5 min and stored in a sealed container for 24 h. Then, the prepared soil and cement were slowly added to the bentonite slurry and mixed again for 5 min. A slurry mixing bucket was used for the mixing tool. Subsequently, the well-mixed slurry was cast into molds (φ50 × 100 mm and 70 × 70 × 70 mm) with different specifications. Then, the specimens were placed in a curing environment (humidity >95%, 20 ± 2 °C) for 24 ± 2 h and then demolded and continuously maintained until the test time. A mini-slump bucket was used in the experiment by Fan et al. [35]. The slurry that met the requirements of construction had a collapse between 22–48 mm. Therefore, in this study, the water content of the slurry was controlled to ensure a mini-slump of 35 mm for different slurry ratios, thereby ensuring engineering availability. Table 2 lists the mixture proportions of the studied backfills.

### 2.3. Experimental Test

#### 2.3.1. Fluidity Test

The mini-slump bucket used in this study has an upper diameter of 75 mm, a lower diameter of 100 mm, and a height of 150 mm. Its conversion equation to a standard slump bucket is as follows:

(1) $$S_{sd}=1.9S_{\min i}+59$$

where *S_sd_* represents the standard slump, and *S_mini_* represents the mini slump.

#### 2.3.2. UCS Test

We used a SANS microcomputer-controlled electronic pressure tester for the UCS test with a loading rate of 1 mm/min. The reference standard was ASTM D2166/D2166M-16 [36]. We used cylindrical specimens of φ50 × 100 mm for the UCS test. Each strength test group consisted of three parallel specimens, and the mean value of the test results was calculated.

#### 2.3.3. Permeability Test

We used a TST-55 permeability meter to measure the permeability coefficient of the specimen. We followed the steps specified in GB/T50123-2019 strictly for the permeability test of the specimens [37]. Each strength test group consisted of three parallel specimens, and the mean value of the test results was calculated.

#### 2.3.4. NMR Test

Many experimental tools are available to help us understand the pore structure of SCB backfills [38,39,40], such as mercury intrusion porosimetry (MIP), nitrogen adsorption and desorption (NAD), and NMR. It is worth noting that conducting the MIP and NAD tests require drying the specimens, which significantly affects the pore structure of the bentonite-containing samples. In contrast, NMR testing can be performed for saturated SCB backfill samples and is a more appropriate testing tool [41,42].

The relationship between relaxation time (*T*_2_) and pore structure in NMR tests can be illustrated by the following equation:

(2) $$\frac{1}{T_{2}}\approx \rho_{2}\left(\frac{S}{V}\right)=\frac{F_{s}\rho_{2}}{r}$$

where *r* is the pore radius (μm), *T*_2_ is the transverse relaxation time (ms), *ρ*_2_ is the transverse surface relaxation strength (μm/ms), *F_s_* is the pore shape factor (for spherical pores, *F_s_* = 3; for tube bundle pores, *F_s_* = 2), and (*S*/*V*) is the pore surface area to fluid area ratio.

We subjected saturated samples cured for 7 and 28 days to NMR testing using the instrument model Meso-MR23060H. We used cylindrical samples of φ50×100 mm for NMR testing. We prepared one sample for each proportion test.

#### 2.3.5. SEM Test

SEM analysis was undertaken using a Quanta-200. We used a sample smaller than 1 cm^3^. We fixed, dehydrated, and dried the sample first. Then, we sprayed a metal film on the surface of the treated specimen and tested it. We prepared one sample for each proportion test.

**Figure 2:**
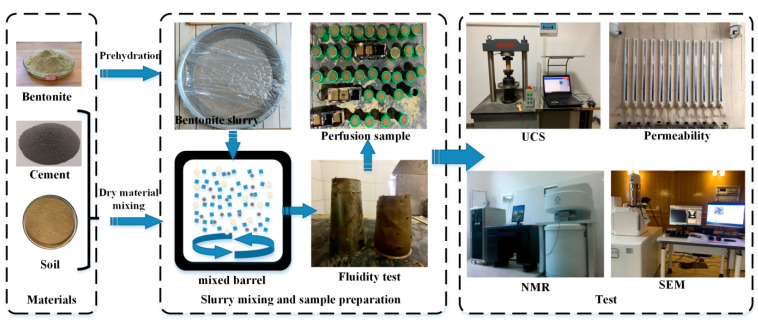
Experimental process.

The experimental process is shown in Figure 2.

## 3. Results

### 3.1. Effect of Bentonite and Cement on Slurry Water Content

**Figure 3:**
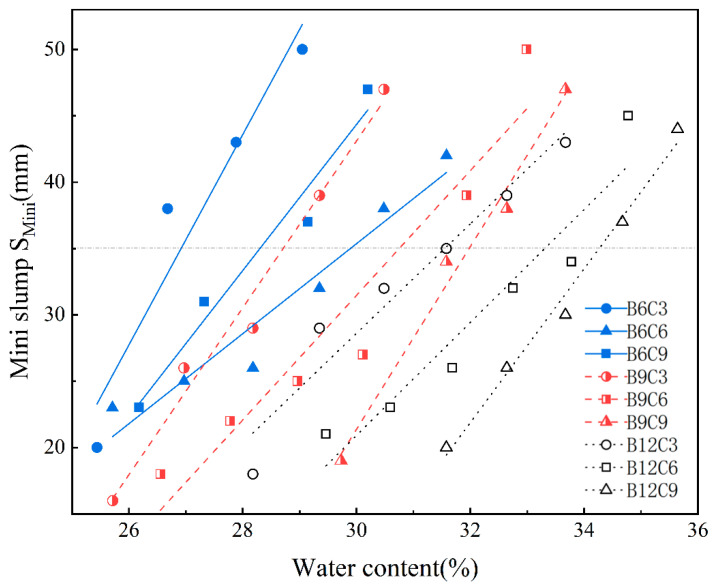
The relationship between slurry water content and slump.

In practice, slurry fluidity significantly affects the quality of the cut-off wall. Higher water content facilitates construction by improving slurry fluidity but impairs the performance of the cut-off wall. Conversely, lower water content enhances the performance of the cut-off wall but increases the risk of local defects in construction due to high viscosity, resulting in poor quality of the cut-off wall. Therefore, only by conducting research based on reasonable fluidity can we balance the relationship between material performance and construction quality. To ensure that the fluidity of all the SCB slurries in the experiment could meet the construction requirements, slump tests with different water contents for each proportion of slurry were performed in pre-experiments using a mini-slump bucket. The relationship between the water content and mini-slump bucket slump for different slurry ratios was shown in Figure 3. Then, the water contents and slumps of the same proportioned slurries were fitted. The results showed that there was a linear correlation between water content and slump, with a correlation coefficient (*R*^2^) ranging from 0.88 to 0.98.

Fan et al. suggest that the optimal range of slurry flowability is 22–48 mm (mini slump). This paper adopts the middle value of 35 mm as the criterion for fluidity [31]. According to the fitting relationship in Figure 3, the corresponding moisture content and actual mini-slump of each group when the mini-slump was approximately 35 mm are shown in Table 3.

**Figure 4:**
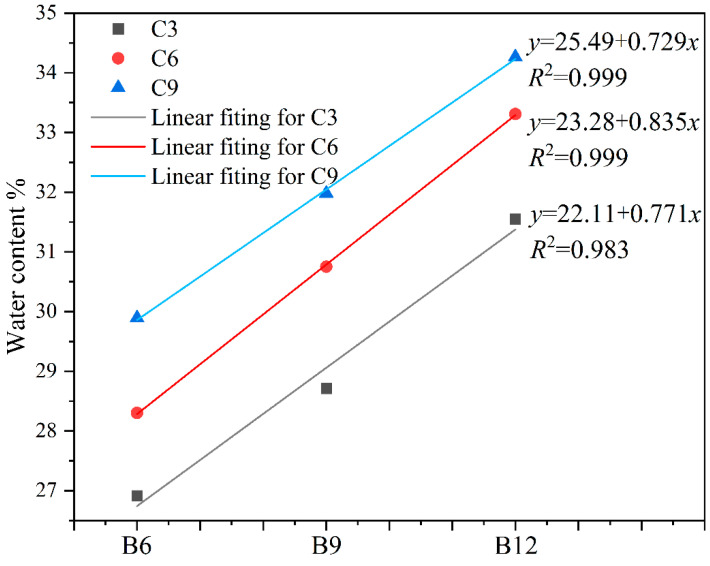
Relationship between water content and bentonite at a slump of 35 mm.

**Figure 5:**
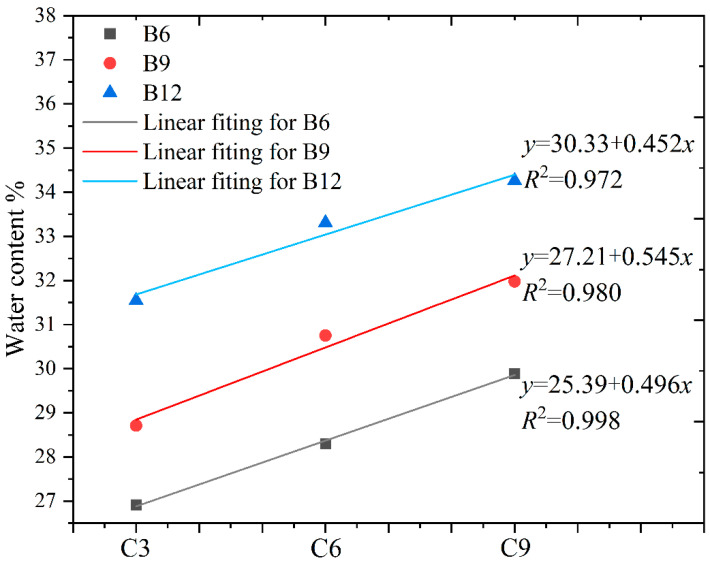
Relationship between water content and cement at a slump of 35 mm.

This study investigated the relationship between the slurry moisture content and bentonite as well as cement at a fixed slump. The variations in the water content with bentonite and cement addition are shown in Figure 4 and Figure 5.

The results indicated that water content significantly increased with increasing bentonite and cement content for a fixed slump. At a slump of 35 mm, slurry with 9% bentonite content had a 7.4% higher water content than the one with 6% bentonite content, and 12% bentonite content had an 8.4% higher water content than the one with 9% bentonite content. Meanwhile, the water content required for slurry to achieve the same slump increased as cement content increased.

We established the relationship between the content of bentonite and cement and the water content by fitting. We illustrated the difference in water demand of the two materials under the same fluidity by using the slope of the fitting line. The fitting calculation showed that, under the condition of the increasing gradient of the same content, the average slope of the bentonite fitting line was 0.778, and that of the cement fitting line was 0.498. According to the fitting results, the water content of the backfill for a fixed slump is more sensitive to the amount of bentonite admixture. The fluidity of the fresh slurry depends on the different water demands of cement and bentonite during initial hydration. Bentonite has a shorter hydration duration than cement due to its unique double-layer structure. The hydration of cement is the exothermic alkali activation reaction of water and cement clinker, which has a longer duration than that of bentonite. Hence, for the same increment, bentonite requires more water than cement in the fresh slurry phase [30,43,44].

### 3.2. Strength Properties

**Figure 6:**
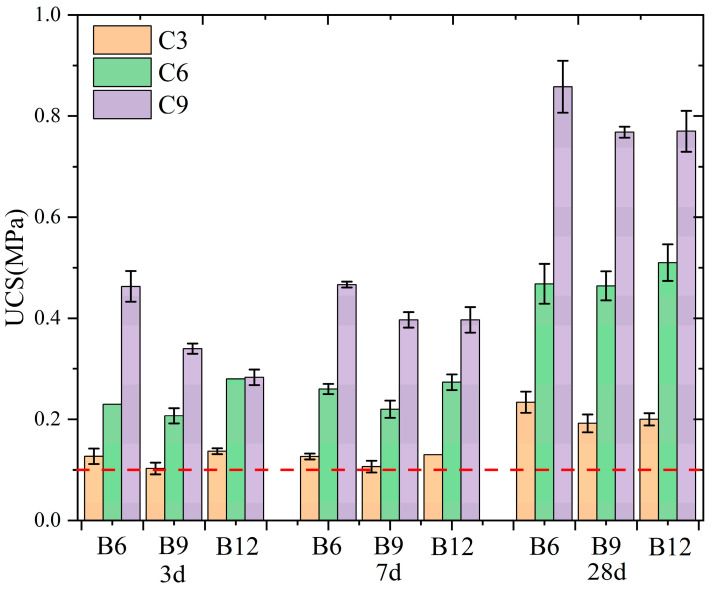
Unconfined compressive strengths of different SCB backfills.

The cut-off wall backfill should not have a very large UCS value to avoid posing an obstacle to land development at a later stage but should have adequate strength to avoid both cracks under loads and localized failure of seepage control. In engineering design practice, the cut-off wall usually has 100 kPa as the basic design strength, and the basic strength requirement is indicated by a red dashed line in Figure 6. As shown in the figure, after 28 days, all the mix ratios met the basic strength requirement (>100 kPa) [32].

The UCSs at various curing ages are shown in Figure 6. The UCS value of the backfill increased with the age of curing. At an early age (before 7 days), the USC of the backfill varied less, whereas the average USC of the backfill at 7 days was 109.5% of that at 3 d. At a later stage of age (7 to 28 days), the average USC of the backfill increased by as much as 187.8%.

The test results indicated that the UCS value of backfill increased as cement content increased at a certain bentonite content. In the B6 group, at different ages, mean UCS values in C6 and C9 were 95.5 and 88.0% higher than the ones in C3 and C6, respectively. In the B9 group, at different ages, mean UCS values in C6 and C9 were 116.3 and 70.0% higher than the ones in C3 and C6, respectively. In the B12 group, at different ages, mean UCS values in C6 and C9 were 123.2 and 32.4% higher than the ones in C3 and C6, respectively. The data showed that the increase in the average strength at each backfill age gradually became larger when the cement was increased from 3% to 6%. At a certain cement content, the UCS of the backfill changed with the bentonite content without exhibiting an obvious pattern. This contradicts the findings of Hui et al. [30] and Wu et al. [45], who reported that the sample strength decreased with the increasing bentonite content. Among these groups, the C3 and C6 groups exhibited a trend of first decreasing and then increasing with the addition of bentonite, and the C9 group exhibited a decreasing trend and then stabilized. There are several reasons for this phenomenon. First, as the bentonite content increases, the moisture content of the backfill increases and results in changes in the pore structure of the backfill. Second, in the hydration process, bentonite and cement compete for water, thereby resulting in different degrees of hydration of the backfill. Overall, the increased bentonite content at a larger cement content in the backfill directly affects the degree of hydration of the cement.

### 3.3. Permeability

**Figure 7:**
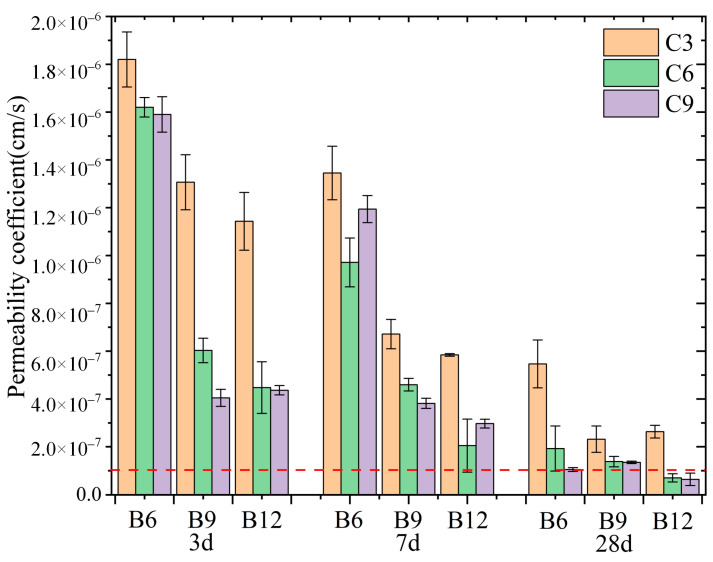
Permeability coefficients of different SCB backfills.

As shown in Figure 7, as curing time increased, the backfill permeability coefficient significantly decreased. When the bentonite content was 6% (B6), the backfill permeability coefficient at an early age was larger; however, its decline with time was more obvious. When the bentonite content was 9% and 12% (B9 and B12 groups, respectively), the permeability coefficient of the backfill reached a smaller value at 3 d and changed less with curing time. A common criterion for the cut-off wall is a permeability coefficient of <1 × 10^−7^ cm/s after 28 days curing. We indicated the basic permeability coefficient criterion with a red dashed line in Figure 7 [16].

The results showed that the permeability of the backfill reduced with an increase in the cement content at a certain bentonite content. At this time, cement hydration was the main factor causing a decrease in the permeability coefficient. The higher the cement in the backfill, the more cementation products formed in the hydration reaction. These hydration products filled the pores of the soil particles, which made the backfill more compact and led to a decrease in the permeability coefficient. However, the decrease in the permeability coefficient gradually slowed with an increase in the cement content [44]. The permeability coefficient of group C6 was, on average, 45.37% lower than that of group C3, and the permeability coefficient of group C9 was, on average, 6.22% lower than that of group C6.

When the cement content in the backfill was fixed, the overall permeability coefficient of the backfill decreased with an increase in the bentonite. Ca^2+^ ions in the hydrated cement initially formed calcium hydroxide (C-H), which subsequently reacted with the silicate and aluminate in the bentonite, forming abundant hydration products [46]. This densifies the SCB structure and decreases the SCB permeability. The permeability coefficient of group B9 was, on average, 43.72% lower than that of group B6, and the permeability coefficient of group B12 was, on average, 23.22% lower than that of group B9.

### 3.4. NMR Test

The NMR T_2_ value can be representative of the pore size in the case of a saturated SCB backfill. The areas of the different peaks in the NMR T_2_ spectra can represent the pore volumes of different pore sizes in the SCB backfill [47].

To investigate the micro-porosity of SCB specimens, we chose the specimens of C3 (B6, B9, and B12) and B9 (C3, C6, and C9) cured for 7 d and 28 days for NMR testing. A previous study showed that the peak area of the T_2_ spectrum collected by the NMR test reflects the amount of physically bound water in the sample [48]. Thus, the first peak can be considered as a signal generated by the adsorbed water representing small pores, and the second peak is a signal generated by free water representing large pores.

**Figure 8:**
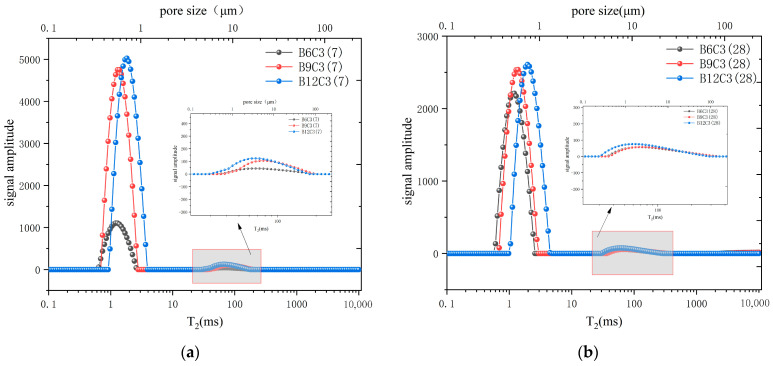
NMR T_2_ spectral distribution of SCB samples incorporating different contents of bentonite. (**a**) The test results after 7 days of curing. (**b**) The test results after 28 days of curing.

As shown in Figure 8a, peak 1’s area increased as the bentonite content increased in the SCB backfill, and its peak center shifted from left to right at 7 days. This indicated that as the bentonite content increased, more adsorbed water could be accommodated in the microstructure of the SCB backfill [49]. Furthermore, this implied that the number of small pores in the SCB backfill gradually increased along with the structure. Compared with B6 at 7 days, B9 and B12 displayed a broader distribution of peak 2. Moreover, the area of peak 2 increased with the increasing bentonite content in the SCB backfill, and the peak center shifted from right to left with increasing bentonite dosage. This showed that as bentonite was added, the number of large pores in SCB backfill gradually increased, but the size of the large pores tended to decrease, which implied that adding bentonite could optimize the pore structure of SCB backfill. Figure 8b showed the NMR T_2_ spectra of group C3 at 28 days; the trend of the 28 days T_2_ spectra of each group was similar to the one at 7 days as curing time increased. This indicated that hydration products in the SCB backfill of group C3 did not significantly increase as curing time increased, unlike the pore change trend in conventional cementitious materials. This might be because a large amount of free water in the SCB backfill was consumed by bentonite during the curing process and transformed into bentonite interlayer chemistry water, which affected the hydration process of the SCB material.

**Figure 9:**
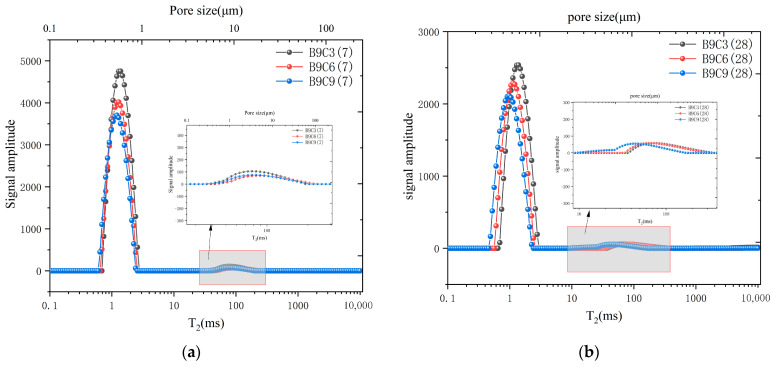
NMR T_2_ spectral distribution of SCB samples incorporating different contents of cement. (**a**) The test results after 7 days of curing. (**b**) The test results after 28 days of curing.

As shown in Figure 9a, the relaxation region of the T_2_ spectrum did not significantly change as cement content increased, initial relaxation time was slightly advanced, and the peak decreased. This implied that the number of small pores in the SCB backfill gradually reduced and that the structure of small pores was slightly reduced. The relaxation time in Figure 9a showed that the relaxation range of peak 2 on 7 days was similar for group B9 with increasing cement content, and peak strength gradually decreased. This indicated that the structure of the large pores in the backfill did not change as the cement content increased after 7 days of curing, but the number of pores gradually decreased. Figure 9b showed the NMR T_2_ spectrum of group C3 after 28 days. As curing time increased, the T_2_ spectrum morphology of group B9 was significantly different from the one at 7 days. This indicated that the pore structure of small pores in the SCB backfill gradually decreased and that pore number gradually decreased as curing time increased, which indicated that more hydration products were present in the SCB backfill with higher cement content after 28 days of curing, and increased hydration products optimized the structure of small pores in backfill. In the B9 group, the spectral shape of peak 2 at 28 days was slightly different from the one at 7 days. The T_2_ spectrum distribution of peak 2 was wider, and its peak center moved to the left for B9C3 and B9C6, which indicated that the distribution of large pores in low-cement-dosing backfill was wider as curing time increased, but, overall, the structure of the large pores showed a decreasing trend. A large quantity of hydration products were continuously produced during curing of SCB backfill. These hydration products filled larger pores within the backfill. The T_2_ spectrum of B9C9 changed more significantly after 28 days of curing. At this time, peak 2 of B9C9 group changed from a single peak to a double half-peak, its initial relaxation time advanced, and its peak center exhibited a more pronounced trend shift to the left, suggesting that higher cement content further reduced the large pore size and optimized the overall large pore structure.

**Figure 10:**
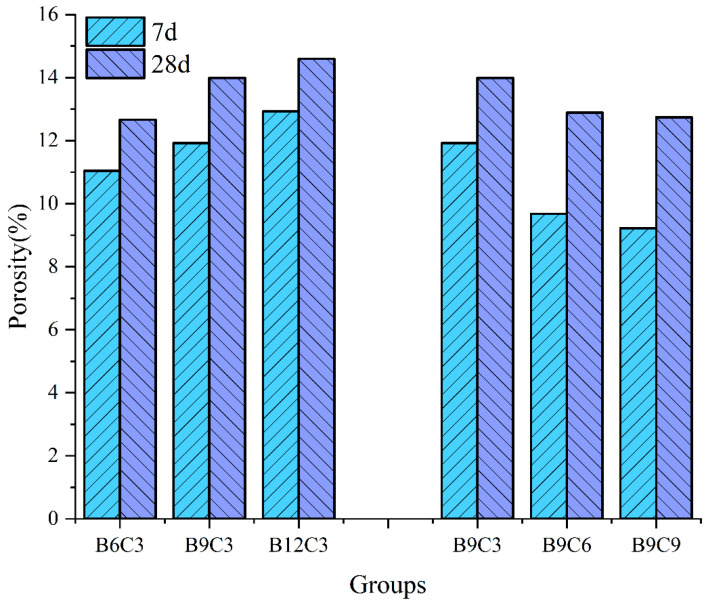
Porosity of SCB samples of different groups.

This subsection presents the effects of different ratios of bentonite and cement on the porosity of the SCB backfill for groups C3 (B6, B9, and B12) and B9 (C3, C6, and C9) as examples. Figure 10 shows that with the increase in bentonite or cement, the porosity of the SCB backfill exhibits an opposite trend, that is, increasing the bentonite content increases the porosity, while increasing the cement content decreases the porosity. This is because more water is required in the backfill with a high bentonite content for a fixed slump of the SCB backfill, which can lead to an increase in the initial water content of the backfill; consequently, this leads to a higher backfill porosity [30,45]. Meanwhile, the porosity of the backfill decreased with an increase in the cement content, but the decreasing trend slowed down. This indicates that adding cementitious materials with a content greater than 6% in group B9 did not significantly reduce the porosity of the backfill.

### 3.5. SEM Observation

**Figure 11:**
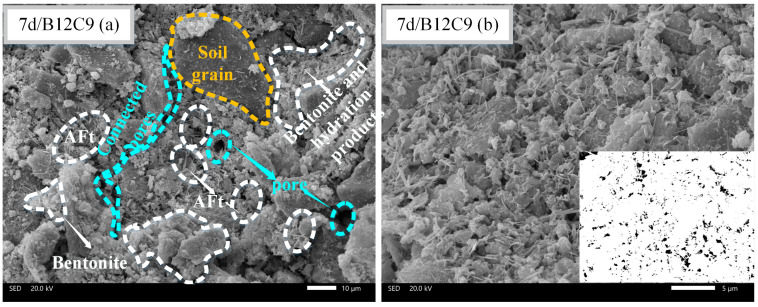
SEM images of B12C9 backfill after 7 days of curing. (**a**) The observation field at 1000× magnification, (**b**) The observation field and binary image at 3000× magnification.

**Figure 12:**
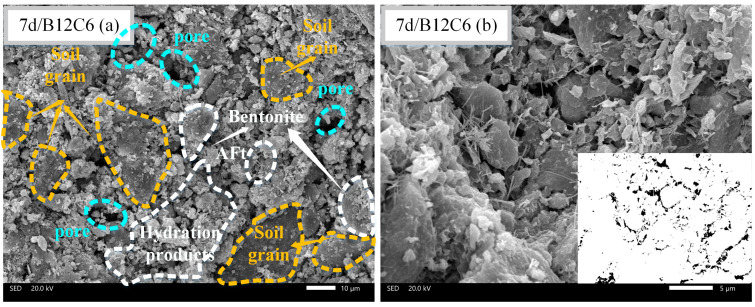
SEM images of B12C6 backfill after 7 days of curing. (**a**) The observation field at 1000× magnification, (**b**) The observation field and binary image at 3000× magnification.

**Figure 13:**
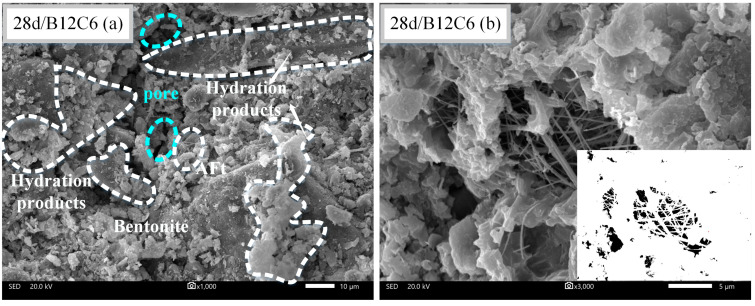
SEM images of B12C6 backfill after 28 days of curing. (**a**) The observation field at 1000× magnification, (**b**) The observation field and binary image at 3000× magnification.

Different backfill micromorphologies can be observed at different curing times. Figure 11, Figure 12 and Figure 13 present the SEM images of B12C9 (7days), B12C6 (7days), and B12C6 (28 days), respectively. The (a) sections in Figure 11, Figure 12 and Figure 13 are micrographs magnified by 1000 times, and the (b) sections are micrographs magnified by 3000 times. Moreover, the b sections in Figure 11, Figure 12 and Figure 13 were binarized to qualitatively examine the variations in their pore frameworks. As shown in Figure 11a, the bentonite is flaky with curly and smooth characteristics. Furthermore, a large number of hydration products in the backfill, such as needle-like ettringite (AFt) and hydrated calcium silicate, are surrounding the perimeter of the soil particles together with the bentonite. These mixed hydration products fill the spaces between the soil particles, leading to an increase in the strength and a decrease in the permeability of the backfills.

Figure 11 and Figure 12 show the SEM images of the B12C9 and B12C6 groups on day 7. From the microscopic morphology, the contents of AFt crystals and hydrated calcium silicate gel in the B12C9 group were significantly higher than those in the B12C6 group. The mixing state of the hydration products and bentonite was better in the B12C9 group. In this case, the flocculated bentonite and hydration products reduce the permeability of the B12C9 backfill by filling the pores between the soil particles (Figure 11b). When the curing time of the B12C6 group reached 28 days (Figure 13a), the fibrous structure of AFt and the amorphous hydrated calcium silicate gel were observed to be more closely combined. Some of the larger pores gradually filled. The strength and impermeability of the backfill were further improved.

For the visualization of the pore structure, SEM images were converted to correspond to the binarized images. It can be seen from Figure 12 that the B12C6 backfill at 7 days contained a large number of coupled pores. However, after increasing the cement content (B12C9), the joint pores gradually changed to small, uniformly distributed pores. Meanwhile, when the curing time of the B12C6 backfill reached 28 days, the smaller pores in the backfill disappeared and the larger pores were gradually filled (Figure 13a).

## 4. Analysis and Discussion

### 4.1. Relationship between Strength and Porosity

Some studies have reported a functional relationship between porosity and strength [50]. We compared the porosity and UCS data of groups C3 and B9 to analyze the relationship between the porosity and strength data of SCB backfills with the same flow conditions.

**Figure 14:**
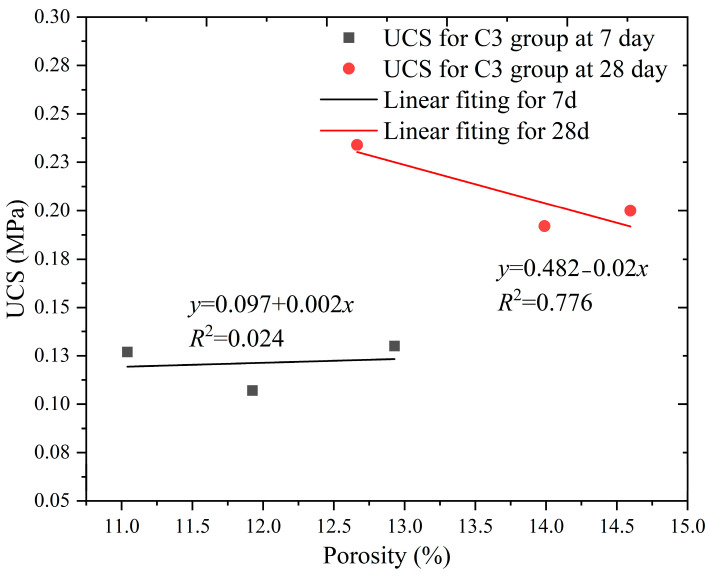
Relationship between UCS and porosity of the C3 group.

As can be seen from Figure 14, with the increase in bentonite, the fitted line for the porosity and strength relationship of the SCB backfill in group C3 after 7 days was approximately horizontal, and the monomer exhibited a fluctuating trend of first decreasing and then increasing. As discussed in Section 3.4, the increase in bentonite in the backfill enhances the slurry water content and increases the total porosity of the material at the early stage of SCB backfill curing. Furthermore, because of the competition between cement and bentonite for water in the slurry, a complex early hydration reaction occurs in the SCB backfill. Because adding a small amount of bentonite to the SCB inhibits the early hydration reaction of cement, it is difficult to produce a large number of hydration products to enhance the strength, and part of the hydrated bentonite fills the micropores, thereby forming a bond; however, the strength enhancement is not obvious. The continued addition of bentonite to the SCB increased the filling of large pores by bentonite and improved the pore structure, whereas the higher water content of the backfill promoted the hydration of the cement and improved the early strength of the SCB backfill to a certain extent. After 28 days, the porosity and strength of the SCB backfill of group C3 had similar trends, but the decreasing trend of the strength with the increasing porosity became noticeable. This is primarily because in the late stage of the hydration reaction, part of the bentonite was affected by ion exchange, which caused adsorption water overflow and resulted in the reduction in the bentonite volume to form pores. At this time, the newly generated hydration products only partially filled the pores, thereby resulting in an increase in the porosity and strength of the backfill with curing time.

**Figure 15:**
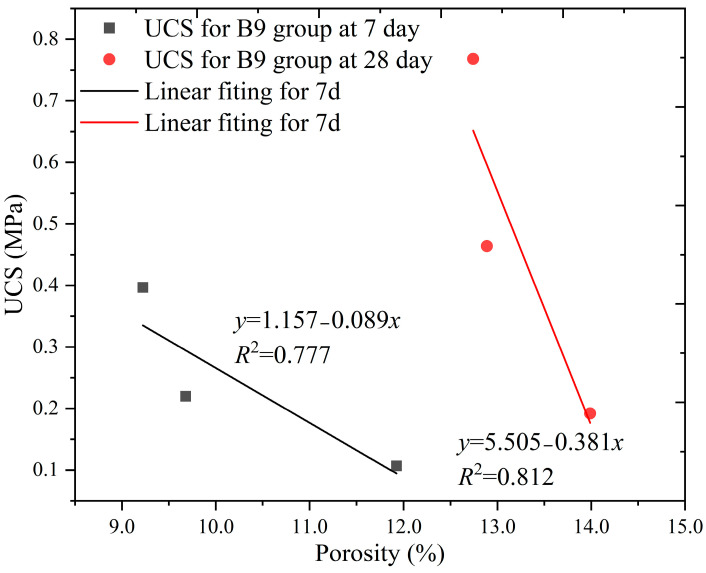
Relationship between UCS and porosity B9 group.

Figure 15 shows the relationship between the porosity and strength of the SCB backfill in group B9. It can be seen from the figure that the strength of the SCB backfill in group B9 at 7 days gradually decreased with increasing porosity. The porosity and strength data of the SCB backfill of group B9 were fitted and found to be well correlated, with *R*^2^ values of 0.777 and 0.812, respectively. The porosity of the specimens decreased faster when the cement content of the backfill of group B9 changed from 3 to 6%; however, the strength decreased more slowly. In comparison, when the cement content of the backfill in group B9 was increased from 6 to 9%, the specimens exhibited a small decrease in porosity and a large increase in strength. This shows that when the bentonite content is 9%, adding 3% or 6% cement can reduce the porosity of the backfill, but it does not improve the pore structure significantly; however, continuing to add it can further improve the pore structure of the backfill. An observation of the slope of the fitted curves for the 7 and 28 days backfills revealed that porosity had a more significant effect on the strength of the SCB material in the later stages of curing. Figure 14 and Figure 15 show that porosity is a strong characteristic of backfill strength only when the filler in the pores of the backfill exhibits bonding and strength properties.

### 4.2. Relationship between Permeability and Porosity

**Figure 16:**
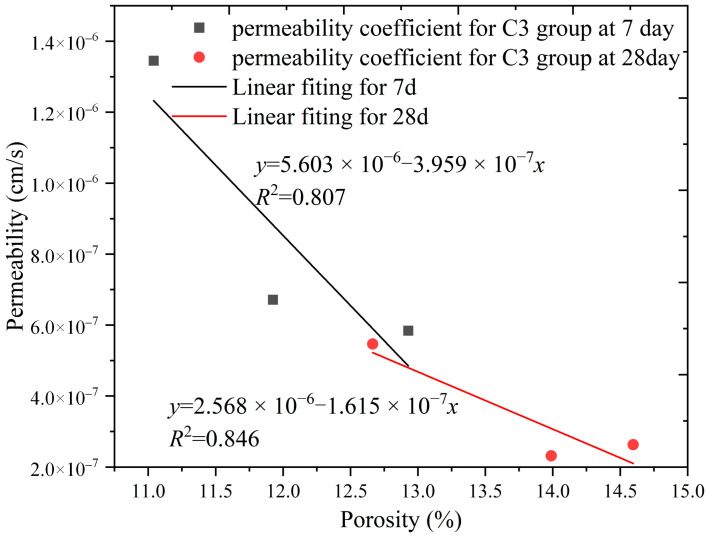
Relationship between permeability and porosity in group C3.

**Figure 17:**
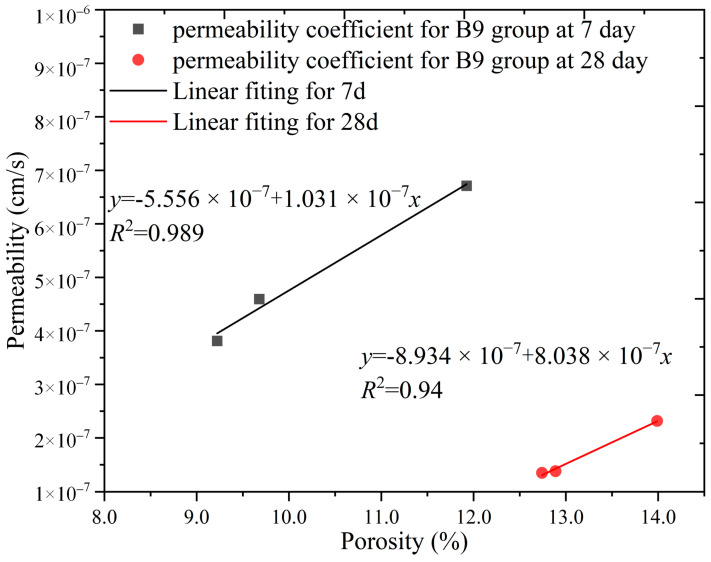
Relationship between permeability and porosity in group B9.

In this study, the porosity and permeability data of the SCB backfill from groups C3 and B9 were analyzed to investigate the relationship between the porosity and permeability coefficient of the backfill for different backfill variables at the same fluidity. The porosity versus permeability coefficients of groups C3 and B9 are plotted in Figure 16 and Figure 17, respectively.

It can be seen from Figure 16 that the porosity increases with increasing bentonite, whereas the permeability coefficient decreases with increasing porosity in specimens of all ages in group C3. The test data were fitted and found to be well correlated, with *R*^2^ values of 0.807 and 0.846. This indicates that the addition of more bentonite increases the porosity of the backfill when the slurry fluidity is the same. However, bentonite optimizes the pore structure of the backfill, increases the volume of small pores, and reduces the permeability of the specimens, which is consistent with the performance of the *T*_2_ spectrum of group C3 in Section 3.4.

Figure 17 shows the relationship between the porosity and permeability coefficient for group B9. It can be seen from the figure that the porosity of the B9 backfill decreased with an increase in the cement content, whereas the permeability coefficient decreased with a decrease in porosity. The porosity and permeability coefficients at 7 and 28 days were fitted and found to be highly correlated, with *R*^2^ values of 0.989 and 0.94, respectively. At 9% bentonite, increasing the cement content from 3 to 6% rapidly reduced the porosity and permeability coefficient. The decrease in porosity slowed down when the cement percentage was increased again, and the decrease in permeability coefficient slowed down. This corroborates the pattern of the *T*_2_ spectra at 7 days for group B9, thereby indicating that the increase in cement content affected both the total pore volume and pore structure.

### 4.3. Pore Throat Analysis

**Figure 18:**
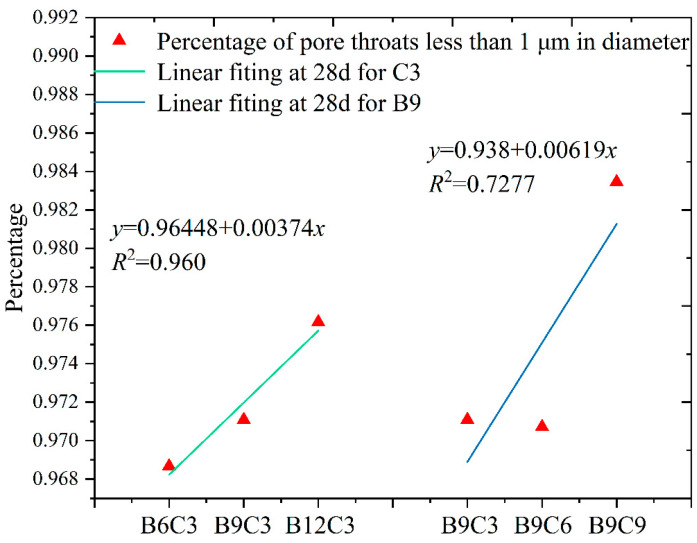
Variation of pore throats in groups C3 and B9.

The pore throat is the inter-pore linkage channel, and the throat size has a significant effect on the permeability of the SCB backfill. An analysis of the pore throat characteristics of the SCB backfill can be used to effectively evaluate the development of seepage channels in the specimens, which can be used to further explain the relationship between the pore and permeability coefficient of the specimens. We obtained the pore throat characteristics of the SCB backfill in the C3 and B9 groups at 28 days by NMR. We defined the pore throat of 0–0.1 μm in the backfill as a micropore throat, a pore throat with a 0.1–1 μm diameter as a small pore throat, and a pore throat with a diameter greater than 1 μm as a medium to large pore throat or fissure. It has been reported that micro- and small-pore throats (<1 μm) in the material are important factors that affect the free flow of water in the pore space [51]. In this subsection, we count the percentage of micro- and small-pore throats in the SCB backfill, which can be used to further analyze the mechanisms of the influences of bentonite and cement on the microstructure and macroscopic properties of the SCB backfill. The percentages of micropores in groups C3 and B9 are shown in Figure 18.

As can be seen in Figure 18, the percentage of the number of micro- and small-pore throats in the backfill of group C3 at 28 days is directly proportional to the amount of bentonite added, and the correlation between the two is strong (*R*^2^ = 0.960). This shows that the addition of bentonite increases the porosity of the backfill and the proportion of micro- and small-pore throats. More micro- and small-pore throats restrict the flow of water in the pores and reduce the permeability of the SCB backfill. This explains why the permeability of the specimens in group C3 in Section 4.2 decreased instead with increasing porosity. Furthermore, this indicates that bentonite mainly has a filling effect on the large pores and inter-pore throats during the curing of the SCB backfill.

The proportion of micro- and small-pore throats in the specimens of group B9 at 28 days also showed a positive correlation with the cement content (*R*^2^ = 0.7277). The percentage of micro- and small-pore throats in the backfill did not change significantly when the cement content was increased from 3% to 6% in group B9. The percentage of micro- and small-pore throats in the backfill increased substantially when the cement content continued to increase. This indicates that small doses of cement can reduce the number of pores in the backfill, but they have no significant improvement on the pore and pore throat structure. Only increasing the cement content further increased the percentage of micro-pore throats.

### 4.4. Engineering Advice

The slurry proportion that meets the actual needs is crucial for the success of cut-off wall construction. The material proportion design on site should consider the construction method, design requirements, and economic benefits comprehensively. First, the slurry workability that meets the site construction conditions should be considered when constructing the cut-off wall using the excavation and backfilling process. The uniform slump control can ensure the smoothness of the construction process and avoid local defects of the constructed cut-off wall. Second, adjust the content of cementitious materials and bentonite based on uniform slump to find the proportion that meets the site requirements. The main parameters to determine the cut-off wall performance are the strength (>100 kPa) and permeability coefficient (<1 × 10^−7^ cm/s). Third, investigate the type of backfill material base. The soil–cement–bentonite (SCB) type of backfill material can use the in situ soil to the maximum extent for construction, reducing the cost of cut-off wall construction and controlling carbon emissions significantly.

This study follows the above process requirements, designs the SCB backfill material proportion using the typical soil in central and southern regions, and suggests a proportion that can be used on-site, satisfying the slump, permeability, and strength requirements. We recommend group B12C9 as the proportion for which the strength and permeability coefficient meet the actual requirements. This provides a reference for the cut-off wall construction design in related areas.

## 5. Conclusions

Based on practical engineering requirements, this study prepared a backfill of SCB with the same fluidity. This study investigated the engineering characteristics and microstructure of SCB backfills with different ratios at the same fluidity through a series of tests, analyzed the effect of bentonite and cement dosages on the engineering characteristics of the SCB backfill, and explored the relationship between the microscopic pore characteristics and engineering characteristics. The following conclusions were drawn.

1. The initial water content of the material increases with the increase in bentonite and cement content. Taking group B9 as an example, under similar fluidity, when the cement content increased from 3% to 9%, the water content of the slurry increased from 28.71% to 31.98%. Similarly, taking group C3 as an example, when the bentonite content increased from 6% to 12%, the water content of the slurry increased from 26.92% to 31.55%. By comparison, it can be seen that bentonite has a greater impact on the initial water content.
2. The strength of backfill soil increased with the increase in cement content. When the cement content increased from 3% to 6%, the increase in strength was greater than that from 6% to 9%. Taking group B12 as an example, the average ucs of groups C6 and C9 were 123.2% and 32.4% higher than those of groups C3 and C6, respectively. With the increase in bentonite content, the strength of backfill soil did not increase significantly. With the increase in cement and bentonite content, the permeability of SCB backfill showed a downward trend, which decreased faster at first and then becomes slower. Generally speaking, due to the interaction between cement and bentonite in the curing process of backfill soil, the change in engineering performance was not proportional to the increase in dosage. Based on the criteria of the strength and permeability coefficient (UCS > 100 kPa, 28 days permeability coefficient <1 × 10^−7^ cm/s), group B12C9 was found to be more suitable for practical engineering applications.
3. When the fluidity was maintained at a constant, the increase in bentonite expanded the pore size distribution range of the large pores in the SCB backfill, reduced the pore size of the large pores, and increased the number of small pores. An increase in the cement content reduced the number of large and small pores and their diameters.
4. The bentonite and cement had opposite effects on the porosity of the SCB backfill. The addition of bentonite promoted an increase in the porosity of the SCB backfill because the initial water content of the SCB backfill with a higher bentonite content was higher. A higher initial water content increased the initial porosity of the backfill, whereas the competition for water at the high content of bentonite reduced the hydration of cement and adversely affected the porosity. The addition of cement promoted a reduction in the porosity of the SCB backfill, which is similar to the porosity performance of general cementitious materials.
5. The strength of the SCB backfill decreased with increasing porosity; however, the C3 group did not perform as well as the B9 group. This shows that most bentonites only play the role of filling pores but do not solidify the backfill structure, whereas cement can fill pores and solidify the structure in the backfill. Furthermore, the porosity of the SCB backfill did not have a general rule for characterizing the permeability. The backfill porosity was inversely proportional to the permeability coefficient when the bentonite content was increased, and the backfill porosity was positively proportional to the permeability coefficient when the cement content was increased.
6. Statistical analyses of the micro- and small-pore throats (<1 μm) in the SCB backfill revealed a positive correlation between the percentage of the number of micro- and small-pore throats and the contents of bentonite and cement in the SCB backfill. It was observed that an increase in the cement and bentonite contents led to a decrease in the pore passage of the SCB backfill, which explains the microscopic phenomenon of increasing porosity and the decreasing permeability coefficient in group C3 backfill. Therefore, the effective method to reduce the permeability of backfill is to add the correct proportion of micro- and small-pore throats.

## Figures and Tables

**Table 1:** Mineralogical properties of the materials.

Class	Bentonite	Cement	Soil
Calcite (%)	3.3	12.7	13.2
Albite (%)	—	—	13.0
Chlorite (%)	—	—	26.5
Quartz (%)	18.3	2.2	26.2
Amphibole (%)	—	—	4.1
Mica (%)	17.3	—	9.1
Plagioclase (%)	—	—	3.7
Dolomite (%)	—	—	4.3
Calcium silicate (%)	—	48.5	—
Larnite (%)	—	14.6	—
Gypsum (%)	—	1.0	—
Anhydrous gypsum (%)	—	5.1	—
Brownmillerite (%)	—	15.5	—
Montmorillonite (%)	43.2	—	—
Feldspar (%)	17.9	—	—

**Table 2:** Mixture proportions for the SCB backfills.

Test Number	Bentonite (wt%)	Cement (wt%)	Soil (wt%)
B6C3	6%	3%	91%
B6C6		6%	88%
B6C9		9%	85%
B9C3	9%	3%	88%
B9C6		6%	85%
B9C9		9%	82%
B12C3	12%	3%	85%
B12C6		6%	82%
B12C9		9%	79%

**Table 3:** Water content and mini-slump of SCB slurries.

Test Number	Water Content (wt%)	Mini Slump (mm)
B6C3	26.92	35.8
B6C6	28.30	34.5
B6C9	29.89	35.3
B9C3	28.71	36.1
B9C6	30.75	34.0
B9C9	31.98	36.0
B12C3	31.55	35.2
B12C6	33.31	34.3
B12C9	34.26	34.8

## Data Availability

The data presented in this study are available on request from the corresponding author. The data are not publicly available due to the requirements of the research project.
